# Birth-Related Subdural Hemorrhage in Asymptomatic Newborns: Magnetic Resonance Imaging Prevalence and Evolution of Intracranial and Intraspinal Localization

**DOI:** 10.3390/tomography11050058

**Published:** 2025-05-20

**Authors:** Davide Turilli, Leandra Piscopo, Alberto Dessì, Claudia Pinna, Liala Mirella Fattacciu, Emma Solinas, Ilaria Conti, Stefania Tamburrini, Giacomo Sica, Michele Klain, Salvatore Masala, Mariano Scaglione

**Affiliations:** 1 Department of Medicine, Surgery and Pharmacy, University of Sassari, 07100 Sassari, Italy; davideturilli@gmail.com (D.T.); dessi.alberto@protonmail.com (A.D.); claudia.pinna@aouss.it (C.P.); lialafattacciu@gmail.com (L.M.F.); solinas.emma@gmail.com (E.S.); i.conti@studenti.uniss.it (I.C.); samasala@uniss.it (S.M.); mscaglione@uniss.it (M.S.); 2 Ospedale del Mare-ASL NA1 Centro, Via Enrico Russo, 11, 80147 Napoli, Italy; tamburrinistefania@gmail.com; 3 Department of Radiology, Monaldi Hospital, 80131 Naples, Italy; giacomo.sica@ospedalideicolli.it; 4 Department of Advanced Biomedical Sciences, University of Naples “Federico II”, 80131 Naples, Italy; micheleklain@libero.it

**Keywords:** asymptomatic newborns, neonatal birth-related intracranial subdural hemorrhages, multiparametric magnetic resonance imaging, central nervous system

## Abstract

**Background**: Neonatal birth-related intracranial subdural hemorrhages (SDHs) represent a form of bleeding inside the skull that occurs in newborns. This condition includes the extravasation of blood both in the encephalic parenchyma and in the extra-axial spaces. Recent studies have shown that SDH and particularly post-traumatic birth-related hemorrhages represent a frequent occurrence, but they are often asymptomatic. The gold standard for the diagnosis and follow-up of patients with SDH is multiparametric Magnetic Resonance Imaging. The aim of this study is to describe our experience by reporting several cases of SDH with different distribution and Central Nervous System involvement by the MRI of this pathology in infants up to 30 days of age. **Methods**: We analyzed the age and sex of the patients included in this study, the localization of SDH in different CNS areas, and their frequency using distribution plots and pie charts. **Results**: About the analysis of the SDH locations in the 32 patients, the most common location was the cerebellum (31/32, 96.9%), followed by parietal and occipital lobes (19/32, 59.4%; 18/32, 56.2%, respectively), falx cerebri (11/32, 34.4%), tentorium cerebelli (10/32, 31.2%), temporal lobes (6/32, 18.7%), and finally cervical and dorsal spine in the same patients (4/32, 12.5%). According to SDH locations, the patients were divided into supratentorial, infratentorial, both, and Spinal Canal. **Conclusions**: Our study confirmed the literature data regarding the neonatal birth-related SDH high frequency, but also allowed us to focus our attention on the rarest spinal SDH localizations with the same benign evolution.

## 1. Introduction

Neonatal birth-related intracranial subdural hemorrhages (SDHs) represent a form of bleeding inside the skull that occurs in newborns. This clinical condition includes extravasation of blood both in the encephalic parenchyma and in the extra-axial spaces (epidural, subdural, subarachnoid, and ventricular) that surround it [1]. Until about forty years ago, they were considered rare and associated with significant morbidity and mortality in the neonatal population [2,3,4,5], with effects that can extend well beyond the infantile period, influencing the future health and quality of life of young patients [6]; this resulted in a wide range of clinical manifestations, which had a decisive impact on recovery prospects and long-term prognoses. However, recent studies have shown that SDH and particularly post-traumatic birth-related hemorrhages represent a frequent occurrence (26–46%) [2,7,8,9], but fortunately, they are often asymptomatic [2], and among the latter, they do not determine certain outcomes, even if they are still responsible for the death of 1–2% of newborns in which significant intracranial damage occurs [10,11,12,13]. Neonatal birth-related SDH is becoming a common finding in Computed Tomography (CT) and especially in Magnetic Resonance Imaging (MRI) scans performed for various indications in neonatal age [11,12].

To date, according to recent guidelines [7,9,14], the gold standard for the diagnosis and follow-up of patients with SDH is certainly the multiparametric MRI, even if ultrasound (US) is also gaining importance due to the good reproducibility of the method and its greater territorial expansion and availability compared to MRI. In particular, these are hemorrhages with only supratentorial or only infratentorial distribution or, more frequently, mixed supra- and infratentorial localization with involvement close to the cerebellar tentorium unilaterally or bilaterally both in the posterior cranial fossa and in the occipital area [2,11], possibly extending along the falx cerebri. Knowledge of this form of hemorrhage and its morphological and topographic characteristics is also fundamental to differentiate them from those caused by abuse [12].

The aim of this study is to describe our experience by reporting several cases of SDH with different distribution and Central Nervous System (CNS) involvement, with particular attention to the first-line imaging of this pathology in infants up to 30 days of age.

In particular, in this retrospective study, we will analyze the baseline characteristics of the enrolled patients, the prevalence of neonatal birth-related intracranial SDH excluding secondary forms, the different SDH locations in the CNS, and the distribution and clinical evolution of SDH, with more attention on the spinal canal localization due to its novelty, compared to the recent literature.

## 2. Materials and Methods

### 2.1. Study Population

The institutional review board approved this study protocol. All the infants up to the age of 1 month who performed an MRI of the brain between February 2018 and January 2024 in the Radiology Department of Surgery, Medicine and Pharmacy of the University of Sassari were included in this retrospective observational study. We obtained the final diagnoses thanks to the complementary role of the imaging and clinical teams. We excluded the infants with suspected and known secondary causes of intracranial hemorrhage, such as intraventricular and/or germinal matrix hemorrhage, parenchymal hemorrhage, dural venous sinus thrombosis, hemorrhagic infarcts, and coagulopathies.

### 2.2. Imaging Protocol

The imaging protocol were obtained using a 1.5 Tesla MRI Tomograph (Philips Achieva D-Stream) and the following MRI sequences were performed: 3-plane localizer; sagittal SE T1 weighted (repetition time/echo time TR/TE 543/16 ms, matrix 256 × 204, number of excitation NEX 2, slice thickness 4 mm, skip 0.4 mm); axial FLAIR T2 (repetition time/echo time TR/TE 11000/140 ms, matrix 256 × 203, number of excitation NEX 1, slice thickness 4 mm, skip 0.4 mm); axial FSE T2 (repetition time/echo time TR/TE 6630/140 ms, matrix 384 × 298, number of excitation NEX 3, slice thickness 4 mm, skip 0.4 mm); axial DWI with ADC map (repetition time/echo time TR/TE 3268/78 ms, matrix 160 × 126, number of excitation NEX 2, slice thickness 4 mm, skip 0.4 mm); axial SE T1 (repetition time/echo time TR/TE 543/16 ms, matrix 256 × 204, number of excitation NEX 2, slice thickness 4 mm, skip 0.4 mm); axial FFE T2* (repetition time/echo time TR/TE 675/23 ms, matrix 256 × 205, number of excitation NEX 3, slice thickness 4 mm, skip 0.4 mm); and coronal FSE T2 (repetition time/echo time TR/TE 6499/140 ms, matrix 384 × 298, number of excitation NEX 3, slice thickness 4 mm, skip 0.4 mm). It was not possible to acquire susceptibility-weighted sequences (SWI) as they were not available. All the MRI examinations were performed with anesthesiologist assistance, and in no case was a paramagnetic contrast medium used. The MRI was analyzed by the consensus agreement of two expert neuroradiologists with 19 and 3 years of experience, respectively; these expert readers showed an excellent level of inter-reader agreement for all the MRI parameters. The positive cases of SDH were categorized based on detailed assessments of clinical and radiologic data. The retrospective cohort of this study was defined by the exclusion criteria, determined by the absence of secondary causes of intracranial hemorrhages.

### 2.3. Statistical Analysis

The Statistical Analysis was performed using the JASP software (0.19.1.0). Continuous data are expressed as mean ± standard deviation and categorical data as a percentage. We analyzed the age and sex of the patients included in this study, the localization of SDH in different areas of the CNS, and their frequency using distribution plots and pie charts. The normality of continuous variables was tested using the Shapiro–Wilk test, confirming that age was normally distributed in both males and females (*p* > 0.05). This allowed the use of a Student’s *t*-test, which revealed a significant difference in mean age between males and females (*p* = 0.0001).

## 3. Results

**Figure 1:**
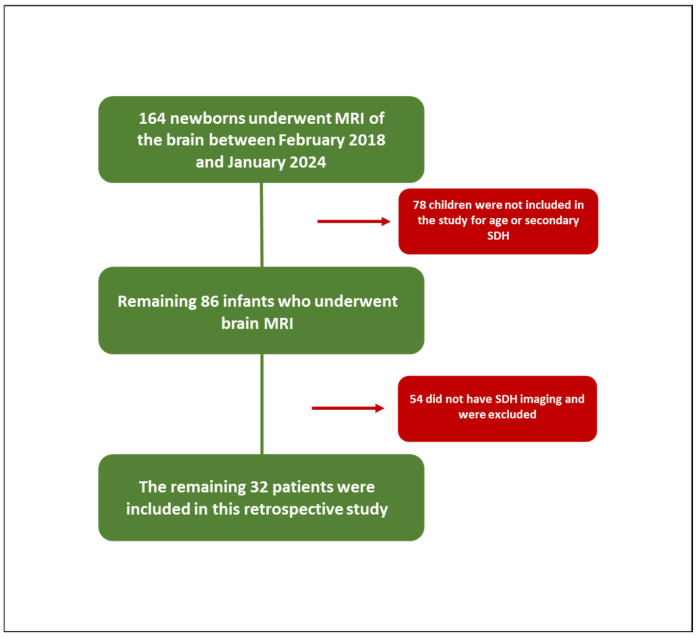
Study population.

A total of 164 newborns underwent an MRI of the brain between February 2018 and January 2024 in the Radiology Department of Surgery, Medicine and Pharmacy of the University of Sassari. The main clinical indications for this protocol imaging were suspected hypoxic–ischemic encephalopathy, hydrocephalus, lethargy, congenital malformations or venous sinus thrombosis, prematurity, non-accidental trauma, trauma, and persistent vomiting. Of these, 78 children were not included in the study because they were 1 month and 1 day old or older or because they were affected by secondary intracranial hemorrhages, and therefore, not related to birth. Of the remaining 86 infants who underwent brain MRI, 54 did not have SDH imaging and were, therefore, excluded from the study, while the remaining 32 patients were included in this retrospective study (Figure 1).

The baseline characteristics of the patient population are summarized in Table 1.

**Figure 2:**
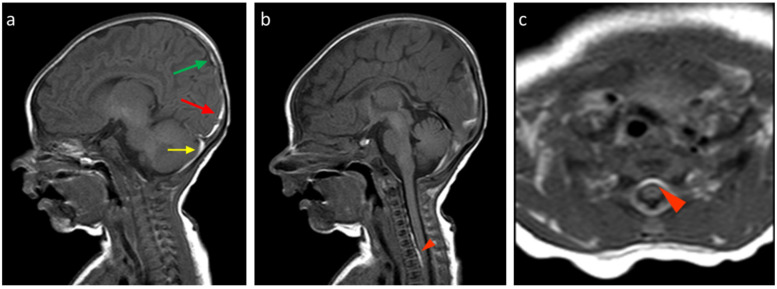
Birth-related SDH in a 9-day-old infant born spontaneously: (**a**) Right paramedian sagittal plane T1-weighted SE sequence—the presence of subdural hematomas with occipital (red arrow), parietal (green arrow), and cerebellar (yellow arrow) distribution. (**b**) Midsagittal plane T1-weighted SE sequence—similar finding in the spinal canal anteriorly in the cervico-dorsal tract (arrowhead). (**c**) SE T1-weighted sequence in the axial plane passing through the distal cervical tract—confirmation (arrowhead).

**Figure 3:**
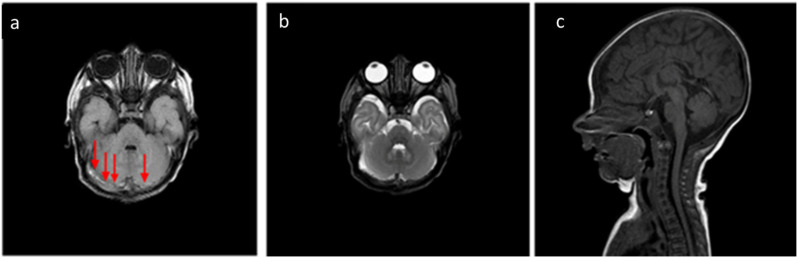
In the same patient in Figure 2, (**a**) FLAIR T2-weighted sequence in the axial plane at the level of the posterior cranial fossa—the subdural hematomas described in the bilateral retrocerebellar region (solid arrow) also appear hyperintense in this sequence, as in the late subacute phase (extracellular methemoglobin). (**b**) FSE T2-weighted sequence on the axial plane at the same 3a level—the known hematomas are difficult to appreciate in relation to the spontaneous hyperintensity. (**c**) SE T1-weighted in the midsagittal plane—the resolution of the hematomas described in Figure 2 and Figure 3 is observed in the follow-up examination.

**Figure 4:**
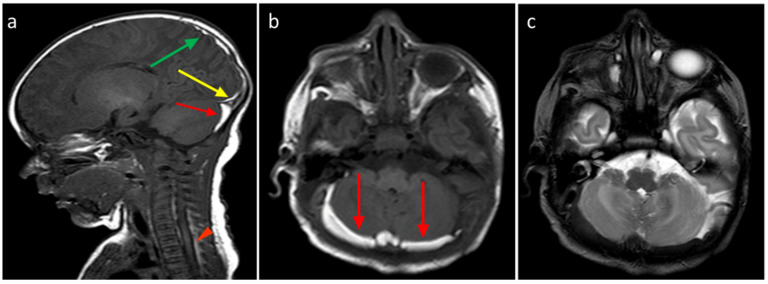
(**a**) Birth-related SDH in a 2-day-old infant born spontaneously. Right paramedian sagittal plane SE T1-weighted sequence—panoramic view of multiple subdural hematomas showing occipital supratentorial (yellow arrow) and parietal (green arrow) and cerebellar infratentorial (red arrow) distribution but also in the spinal canal anteriorly and posteriorly in the cervico-dorsal tract (arrowheads). (**b**) Axial T1-weighted SE sequence passing through the posterior cranial fossa—the infratentorial hematoma shows a development posterior to the vermis and the cerebellar hemispheres, with a greater thickness on the right (arrows). (**c**) FSE T2-weighted sequence on the axial plane at the same level as (**b**) (**b**)—the hematoma shows a hypointense signal, less easily appreciable (arrows), but which, in relation to the hyperintense appearance in T1, allows us to date the hematoma.

**Figure 5:**
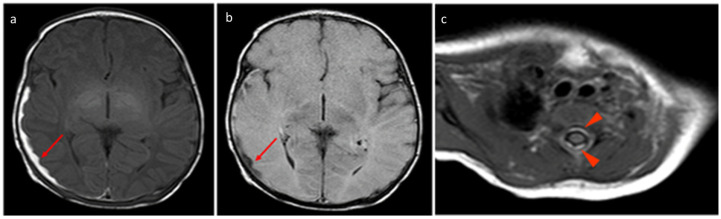
In the same patient in Figure 3, birth-related SDH in an 11-day-old infant born spontaneously:a Axial SE T1-weighted sequence at the level of the basal ganglia—subdural hematoma with occipital and temporal distribution (arrow). (**b**) FLAIR T2-weighted sequence on the axial plane at the same level as in Figure 4a.The hematoma appears hypointense (arrow) and, considering the hyperintensity on T1, it can be dated as early subacute (intracellular methemoglobin). (**c**) SE T1-weighted sequence on the axial plane passing through the distal cervical tract confirms (arrowhead) what was described in Figure 4a.

About the analysis of the SDH locations in the 32 patients which was the object of the study (Table 2, Table 3 and Table 4), the most common location was the cerebellum (31/32, 96.9%) followed by parietal and occipital lobes (19/32, 59.4%; 18/32, 56.2%, respectively), falx cerebri (11/32, 34.4%), tentorium cerebelli (10/32, 31.2%), temporal lobes (6/32, 18.7%), and finally, cervical and dorsal spine in the same patients (4/32, 12.5%, Figure 2, Figure 3, Figure 4 and Figure 5).

According to SDH locations, the patients were divided into supratentorial, infratentorial, both, and spinal canal (Table 5). Twenty-six patients (83%) showed simultaneous and/or bilateral co-involvement of multiple CNS areas. Cerebellum involvement occurred in almost all patients (31/32, 96.9%) except in one 3-day-old infant. In the cerebellum, bilateral involvement was observed in 78% (18/23) of males and 44.5% (4/9) of females. Parietal and occipital SDH were predominantly bilateral in both genders (68.7% and 100%, males and females, respectively), whereas tentorial hemorrhages were more frequently unilateral. The SDH of the falx cerebri and temporal area were more often bilateral in females, while males exhibited a higher prevalence of unilateral involvement in these areas.

**Figure 6:**
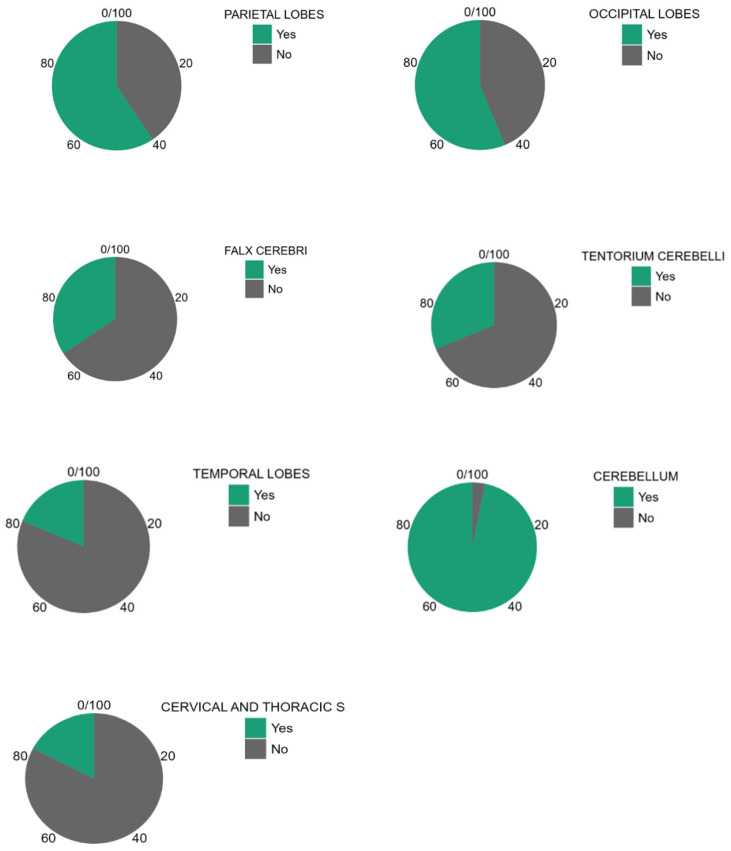
Pie charts according to CNS-involved area.

The pie charts in Figure 6 show the different study populations according to the CNS-involved area.

The thickness of the hematomas varied from approximately 1 to 10.5 mm for infratentorial locations, from 1 to 5 mm for supratentorial ones, and from 1 to 2 mm for intraspinal involvement.

## 4. Discussion

Neonatal birth-related intracranial subdural hemorrhages represent a complex medical challenge. They can be the result of numerous inherited or acquired pathological conditions, although their causes often remain unknown [15].

Among the various forms, we mention ischemic–hemorrhagic vascular, malformation vascular, related to prematurity, linked to a hemorrhagic diathesis, genetic, infectious, neoplastic, and post-traumatic [15]. They have always been considered a rare pathological entity compared to the analogous forms that occur in adults; however, their real incidence has been largely underestimated, as recent studies have demonstrated the existence of a very frequent birth-related SDH post-traumatic form, asymptomatic in most affected newborns [2,15]. From the literature data, it emerges that the main risk factor associated with a greater incidence of birth-related SDH is represented by vaginal birth, whether spontaneous or assisted [7,8,14]; therefore, some authors consider such hemorrhages a common consequence of a normal vaginal birth [8]. Most of these hematomas are small and thin, and they mostly reabsorb within a month; therefore, infants older than 30 days were excluded from this study [2,7,9,14]. As already mentioned, ultrasound (US) has also become a method of the first line in the neonatal field, and in the approach to the study of the brain, for its advantages such as harmlessness, the wide availability of equipment, and the facile execution. It, therefore, represents a new standard approach to detecting germinal matrix hemorrhage in the preterm newborn. Furthermore, its validity in the study of SDH, especially of the posterior fossa, has been demonstrated [7]. Therefore, a high-resolution US study may represent an initial imaging step, but undoubtedly, the gold standard in the diagnostic approach of this patient niche is represented by the multiparametric MRI [1,7,9,14]. Acute cases of SDH are the most insidious to detect [9]; in this group, the acquisition of gradient echo sequences and/or magnetic susceptibility-weighted imaging is fundamental [9,14,16], while in the subacute cases, which in our series were the clearly prevalent ones considering the timing of the exam, without any doubt, the most sensitive sequences were the T1-weighted ones, which we acquired on the sagittal and axial planes [7]. When multiples show similar dating [1,8], it, in any case, correlates with the date of birth.

The hematomas in our series were, in agreement with the literature, small and homogeneous [7], and those subjected to follow-up resolved spontaneously mostly by the thirtieth day of life [7]. Unlike the forms that affect adults, cases that have evolved into chronic forms are not documented [7,9,15,17]. On the other hand, while the most significant traumatic forms show signs of themselves in a few hours/days [14], these benign forms remain asymptomatic, and various studies have not demonstrated significant differences in clinical outcomes when compared with the normal population [6,14,18]. In this retrospective study we did not enroll asymptomatic newborns but only those with various clinical pictures, which did not change with the subdural hematoma’s resolution, and therefore, are not responsible for the symptoms. In particular, the MRI study protocols and clinical evaluations were the same both at the time of diagnosis and at the follow-up.

The sample covered by our study is mostly represented by males (23/32, 71.9%); however, in the literature, no differences in disease presentation related to the sex of the young patient are described. In the recent literature, the CNS areas most involved are the posterior structures, both infra- (cerebellar) and supratentorial (occipital–parietal) [1,2,7,8,9,14,19,20,21]. This type of distribution suggests that these forms of bleeding derive from tributary vessels of the venous sinuses of the dura mater within the folds of the same [18,19]. On the other hand, SDH associated with child abuse trauma is frequently located along the interhemispheric fissure or the cerebral convexities [8,22,23,24].

The absence of hematomas with exclusive infratentorial localization is reported by Rooks et al. [7], who also reveal that all their patients had a supratentorial involvement. On the other hand, a case with only supratentorial localization is described by Whitby et al. [9], which describes six cases out of nine with exclusive infratentorial involvement. Rooks et al. [7] also report 1/4 of newborns with a single site and 2/3 of cases with two or more localizations. In our study, the SDH predominant localization was infratentorial, compatible with the cerebellum localization (31/32, 96.9%) with a single un-affected case and with nine cases with exclusively infratentorial distribution, while no cases with only supratentorial localization were detected. In the latter localization, the most frequent distribution was the parietal one (19/32, 59.4%), followed by the occipital one (18/32, 56.2%), while SDH of the temporal lobe, tentorium cerebelli, and falx cerebri were rarer (6/32, 18.7%; 10/32, 31.2%; and 11/32, 34.4%, respectively). Our case series has highlighted a clear prevalence of male patients (23/32, 71.9%).

The most frequent localization was the cerebellum (31/32, 96.9%), with a single unaffected case, while no cases with only supratentorial localization were detected. The spinal canal represented the least involved site (4/32, 12.5%), with both cervical and dorsal involvement in the same patients. In particular, we observed very thin hematomas with anterior and/or posterior distribution. These cases were associated with a simultaneous involvement of both supratentorial and infratentorial CNS structures. After a month, these four spinal SDH patients were re-evaluated, and a complete resolution with restitutio ad integrum was reached.

Noteworthy is the fact that this disease presentation is very rare, and to date, no cases of SDH with involvement of the spinal canal have been described in the literature [14].

In particular, in newborns, using sagittal MRI sequences of the brain with a field of view (FOV) of 180 mm, it is possible to explore the entire cervical spine and the mid-proximal sectors of the dorsal spine. Therefore, spinal hematomas can be detected shortening the exam acquisition time and sedation, and consequently lowering the costs in order to make this exam more accessible and applicable in different countries.

The spinal SDH forms, in our limited sample, had the same disease evolution in prognostic terms as the classical supra- and infratentorial forms of SDH. It is, therefore, essential to know how to differentiate the primary from the secondary forms of SDH. The resulting treatment is completely different: while in the first case, in most patients, it is self-limiting, in the second, it depends on the underlying pathology.

## 5. Conclusions

Our study confirmed the literature data regarding neonatal birth-related intracranial subdural hemorrhages of high frequency but also allowed us to focus our attention on the rarest spinal SDH localizations with the same benign evolution.

The limitation of this study is certainly represented by the low sample size, which must, however, be contextualized with the limited literature on this very interesting topic.

## Figures and Tables

**Table 1:** Baseline characteristics of study population.

	All Patients *n* = 32
Male gender, *n* (%)	23 (71.9%)
Female gender, *n* (%)	9 (28.1%)
Age male gender (days *± sd*)	8.5 ± 4.5
Age female gender (days *± sd*)	12.4 ± 3.2

**Table 2:** Location of birth-related subdural hemorrhage in supratentorial compartment.

Supratentorial SDH	Count	Percentage
Parietal lobes	19/32	59.4%
Occipital lobes	18/32	56.2%
Falx cerebri	11/32	34.4%
Tentorium cerebelli	10/32	31.2%
Temporal lobes	6/32	18.7%

**Table 3:** Location of birth-related subdural hemorrhage in infratentorial compartment.

Infratentorial SDH	Count	Percentage
Cerebellum	31/32	96.9%

**Table 4:** Location of birth-related subdural hemorrhage in intraspinal compartment.

Intraspinal SDH	Count	Percentage
Cervical and Dorsal Spinal	4/32	12.5%

**Table 5:** Distribution of birth-related subdural hemorrhage.

Birth-Related SDH	Count	Percentage
Supratentorial	23/32	59.4%
Infratentorial	31/32	56.2%
Both	22/32	34.4%
Spinal Canal	4/32	31.2%

## Data Availability

The data presented in this study are available upon request from the corresponding author. The data are not publicly available due to privacy restrictions.
